# Acute Physiologic and Chronic Health Evaluation II and Simplified Acute Physiology Score 3 Scores as Predictors of Mortality in Cardiac Surgery

**DOI:** 10.21470/1678-9741-2025-0098

**Published:** 2026-09-10

**Authors:** Tatiani Bellettini-Santos, Rafael Folador Frederico, Silas Nascimento Ronchi, Jackelyne Lopes Silva, Heloísa Faltz Pimentel, Michelle Lima Garcez

**Affiliations:** 1 Graduate Program in Health Sciences, Centro Universitário do Espírito Santo, Colatina, Espírito Santo, Brazil; 2 Graduate Program in Pharmacy, Department of Clinical Analysis, Universidade Federal de Santa Catarina, Florianópolis, Santa Catarina, Brazil

**Keywords:** Cardiac Surgery, Disease Severity Index, Intensive Care Unit, APACHE II, SAPS 3, Heart Disease

## Abstract

**Introduction:**

Acute Physiologic and Chronic Health Evaluation (APACHE) II is an effective tool to predict outcomes in cardiac surgery recovery. The present study aimed to evaluate the efficacy and compare the performance of the APACHE II and Simplified Acute Physiology Score (SAPS 3) scoring tools as predictive indices of mortality in cardiac surgery patients.

**Methods:**

This was a retrospective observational study, evaluating patients over 18 years of age who underwent cardiac surgeries between 2020 and 2023. A receiver operating characteristic curve analysis was conducted to evaluate the sensitivity and specificity of the APACHE II and SAPS 3 tools in detecting intensive care unit (ICU) mortality in critically ill patients. Binary logistic regression was performed to assess the odds of mortality between groups (primary outcome) using data on comorbidities.

**Results:**

APACHE II was efficient in predicting mortality in cardiac surgery (sensitivity = 81%; specificity = 75%) as was SAPS 3 (sensitivity = 75%; specificity = 64%). Binary logistic regression revealed that mortality was significantly associated with comorbidities (chronic obstructive pulmonary disease and non-dialysis-dependent chronic kidney disease) and the type of surgery, with a higher risk in emergency procedures compared to elective ones.

**Conclusion:**

In conclusion, APACHE II and SAPS 3 proved to be efficient tools for predicting mortality in cardiac surgery patients admitted to the ICU. This study contributes to understanding these tools in a regional context and specifically in cardiac surgeries, as well as assessing other predictors that may be important for identifying risk groups and predicting mortality after cardiac surgeries.

## INTRODUCTION

**Table:** 

Abbreviations, Acronyms & Symbols				
APACHE	= Acute Physiologic and Chronic Health Evaluation		IC	= Intensive care
AUC	= Area under the curve		ICU	= Intensive care unit
CABG	= Coronary artery bypass grafting		ND-CKD	= Non-dialysis chronic kidney disease
CI	= Confidence interval		OR	= Odds ratio
COPD	= Chronic obstructive pulmonary disease		ROC	= Receiver operating characteristic
df	= Degrees of freedom		SAPS	= Simplified Acute Physiology Score
EuroSCORE	= European System for Cardiac Operative Risk Evaluation		SD	= Standard deviation
Exp (B)	= Exponential B		Sig	= Significance
HF	= Heart failure			

Cardiovascular diseases cause thousands of deaths annually on a global scale, and early diagnosis is an ally in preventing potential complications, playing a fundamental role in their effective management^[^1^]^. The occurrence of dysfunction is due to the slow and gradual obstruction of blood vessels, resulting in the partial interruption of blood flow, commonly known as atherosclerosis. This manifests clinically as stroke, angina pectoris, myocardial infarction, or even sudden death^[^2^]^.

Epidemiological data show that various factors can be associated with heart diseases, including hyperlipidemia, hypertension, diabetes, obesity, smoking, and sedentary lifestyle^[^3^]^. These factors can directly influence the management and prognosis of cardiac surgery patients admitted to intensive care unit (ICU)^[^4^]^.

In this context, although the risk of mortality in cardiac surgeries is, in most cases, assessed using preoperative risk scales, numerous intraoperative conditions can also act as risk factors. Considering the parameters of the body’s functionality, these conditions may be relevant in the relationship between the analysis and, as a result, the prognosis of postoperative mortality outcomes^[^5^]^.

However, in clinical practice, the most widely used scoring systems are the Acute Physiology and Chronic Health Evaluation (APACHE) and the Simplified Acute Physiology Score (SAPS), both classified as ICU scores. However, they lack a dedicated postoperative mortality predictor specifically designed for cardiac surgery patients^[^6^]^. On the other hand, the European System for Cardiac Operative Risk Evaluation (EuroSCORE) II, recommended by Brazilian guidelines for preoperative mortality risk assessment in cardiac surgery (particularly for valvular diseases), is a more specialized and easily applicable tool. Its main limitation compared to APACHE II and SAPS 3 is that it relies only on preoperative data, failing to incorporate physiological variables or the patient’s postoperative clinical condition. Therefore, prognostic scores assess the degree of impairment in organic and physiological functions in critically ill patients by translating clinical and/or laboratory changes, as well as the individual’s medical history^[^7^,^8^]^.

In this context, APACHE II, according to the literature, is the most widely used and oldest prognostic analysis tool. In a study involving cardiac surgery patients, it was demonstrated that this clinical system for disease severity classification offers good performance in predicting mortality and reducing the length of stay in intensive care (IC), highlighting a positive factor associated with the use and efficacy of APACHE II^[^9^]^.

On the other hand, in various studies, the SAPS 3 prognostic index has shown unsatisfactory results regarding mortality prediction in the ICU for patients undergoing exclusively cardiac surgery^[^10^,^11^]^. Several studies argue that the most widely used postoperative prognostic scores, including APACHE II and SAPS 3, were developed to assess the severity index of patients admitted to the ICU based on a general, heterogeneous, patient population. This raises concerns about their representativeness and reliability for all types of patients and specific inherent diseases, which also applies to the subgroup of cardiac surgery patients^[^7^,^12^,^13^]^. Thus, given the importance of evaluating these tools in diverse contexts - including regional settings - this study was conducted on a heterogeneous Brazilian population comprising various ethnic groups (Indigenous and European descendants). This research serves two primary purposes: (1) to assess APACHE II’s and SAPS 3’s applicability in regional cardiac surgery contexts while identifying additional mortality predictors, and (2) to specifically evaluate APACHE II’s reliability as a mortality predictor post-cardiac/cardiovascular surgery. Additionally, we analyze survival time, hospital stay duration, and relevant comorbidities while comparing the discriminatory power (survivor *vs.* non-survivor differentiation) of APACHE II *vs.* SAPS 3.

## METHODS

### Study Population

This is a retrospective observational study. The sample population included patients who underwent coronary artery bypass grafting (CABG), aortic replacement, and/or repair with or without procedures involving the ascending aorta, mitral valve, or their combinations, performed at a hospital in the northwest region of Espírito Santo, Brazil. A total of 433 patients who underwent cardiac surgeries between December 2020 and August 2023 were included in the study. Patients with a hospital stay longer than 24 hours and aged over 18 years were included. Excluded data were from patients whose records were unavailable in the electronic medical records and whose hospital stay was less than 24 hours due to discharge, death, or transfer. The project was approved by the Human Research Ethics Committee of the Centro Universitário do Espírito Santo (CEP/UNESC, CAAE nº 70142723.6.0000.5062), technical opinion number 6.573.631.

### Data Collection

Data were collected from electronic medical records and the computerized database (Magma®, Version 4.0) used at a hospital in the northwest region of Espírito Santo, Brazil. Demographic data, length of ICU stay, type of surgery (emergency or elective), severity indices such as SAPS 3 and APACHE II, and data on comorbidities such as non-insulin-dependent diabetes, insulin-dependent diabetes, chronic obstructive pulmonary disease (COPD), alcoholism, chronic atrial fibrillation, hypertension, non-dialysis-dependent renal insufficiency, smoking, obesity, and chronic coronary insufficiency were recorded. The patients were treated as singular data points, regardless of undergoing isolated CABG, valve surgery, or a combined procedure, that was assessed as a single case by the APACHE II and SAPS 3 scoring systems upon ICU admission. The significant associations identified in the univariate logistic regression had odds ratio (OR) > 4. A sample size of approximately 94 participants would be sufficient to detect an OR of 3 with 90% power and 5% significance. Given the final sample of 433 patients and 54 events, the study was adequately powered to detect effect sizes of this magnitude.

### Statistical Analysis

**Fig. 1:**
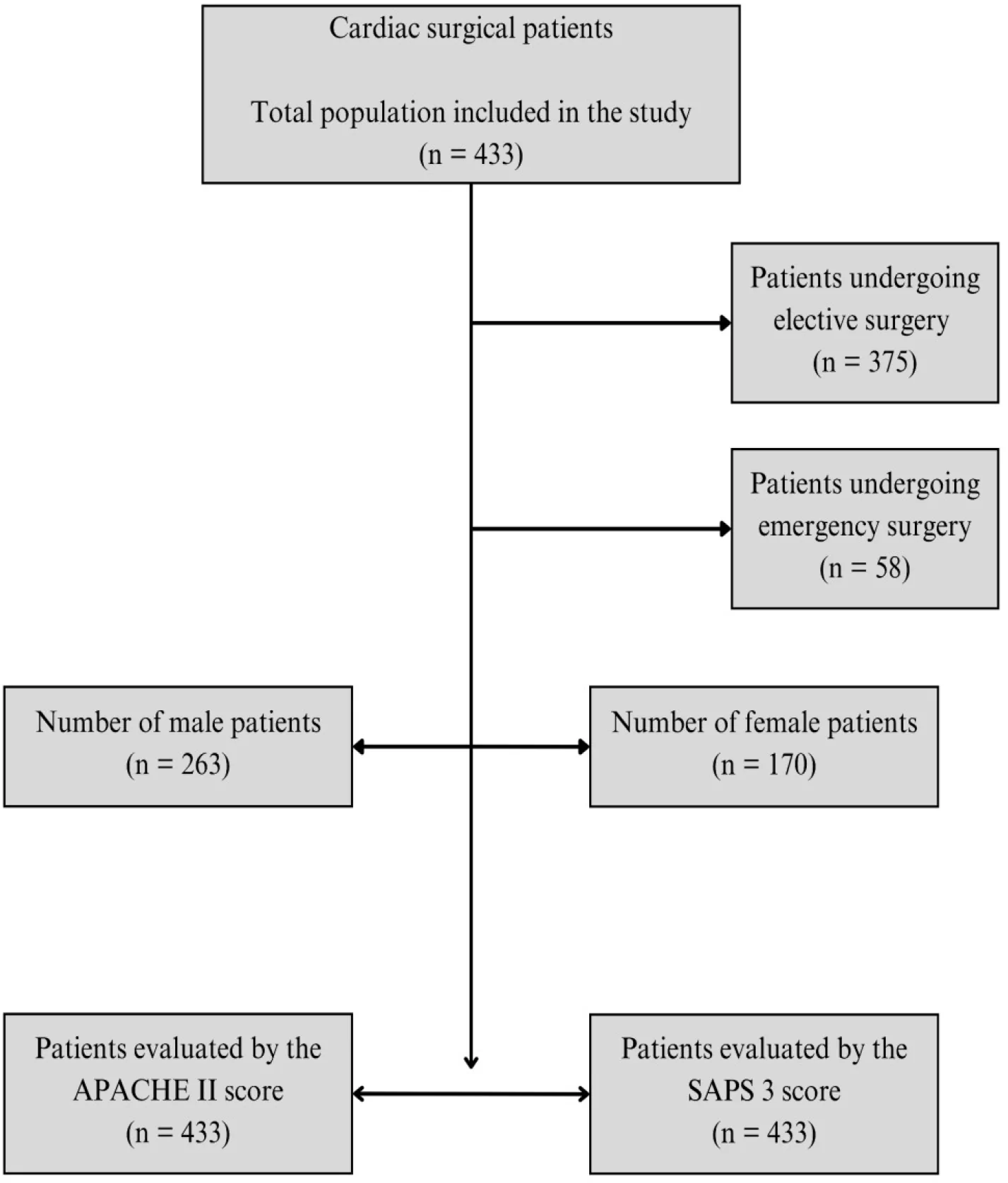
Characteristics of the study population. APACHE=Acute Physiology and Chronic Health Evaluation; SAPS=Simplified Acute Physiology Score.

Statistical analyses were performed using the IBM SPSS Statistics for Windows, version 21.0 (IBM Corp., Armonk, N.Y., USA). To perform the receiver operating characteristic (ROC) curve and DeLong’s test analysis, R Project for Statistical Computing (R version 4.5.1) was used to assess the sensitivity and specificity of the APACHE II and SAPS 3 tools in detecting ICU mortality in critically ill patients (Figure 1). Univariate binary logistic regression was performed to assess the odds of mortality between groups (primary outcome) using data on comorbidities such as non-insulin-dependent diabetes, insulin-dependent diabetes, COPD, alcoholism, chronic atrial fibrillation, hypertension, non-dialysis-dependent renal insufficiency, smoking, obesity, chronic coronary insufficiency, and type of surgery. The results were described using absolute and relative frequencies, mean and standard deviation (SD) for normally distributed data, and median and interquartile range for non-normally distributed data. Data normality was assessed using the Shapiro-Wilk test. The significance level was set at *P* < 0.05.

## RESULTS

A total of 433 patients were included in the study. The median age of the participants was 61 years, with a minimum age of 21 years and a maximum age of 90 years. The average length of ICU stay for these patients was six days, with a minimum of one day and a maximum of 107 days. Males predominated in the sample (59%). Of the 433 patients, 54 died, corresponding to a mortality rate of 12.5%.

**Fig. 2:**
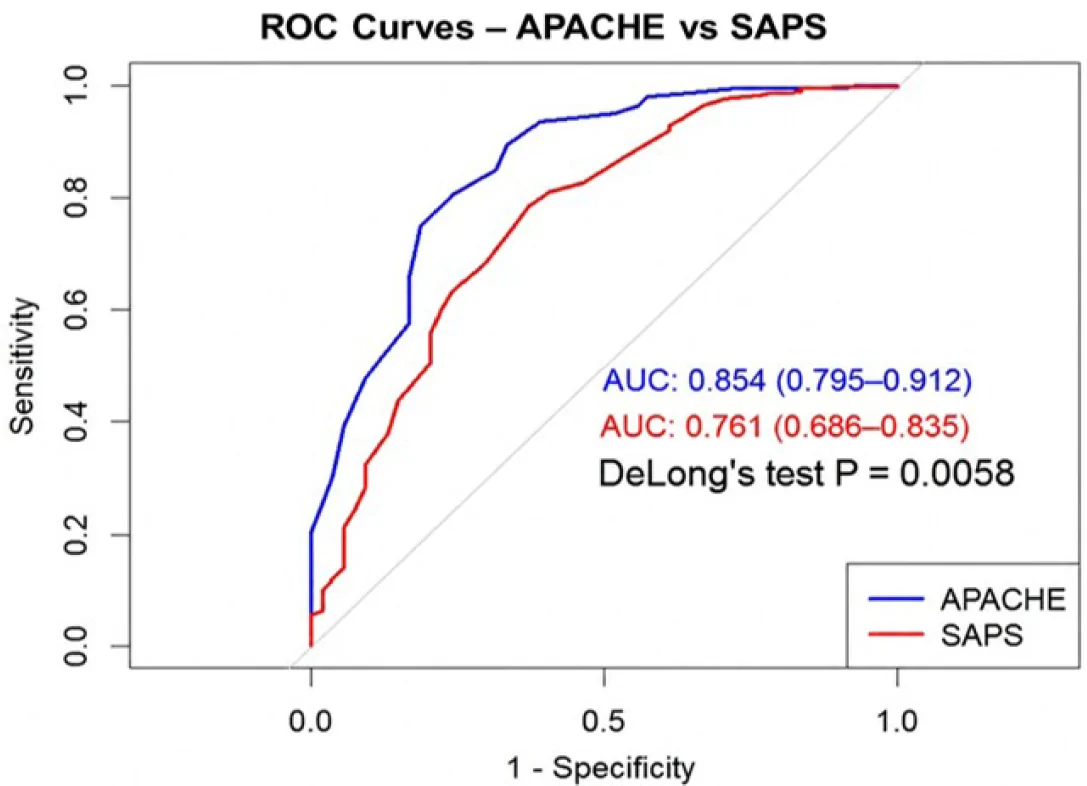
Receiver operating characteristic (ROC) curves comparing the prognostic performance of the Acute Physiology and Chronic Health Evaluation (APACHE) II and Simplified Acute Physiology Score (SAPS) 3 scores in predicting patient outcomes. The APACHE II score (blue line) shows significantly superior discriminative ability, with an area under the curve (AUC) of 0.854 (95% confidence interval [CI]: 0.795 - 0.912), compared to the SAPS 2 score (red line), which had an AUC of 0.761 (95% CI: 0.686 - 0.835). The difference in AUC was statistically significant (DeLong's test, P = 0.0058).

The mean APACHE II score for surviving patients was 12 points (SD = 4.24), and the mean SAPS 3 score was 43.55 (SD = 8.10). For patients who died, the mean APACHE II score was 20.20 (SD = 6.53), and the mean SAPS 3 score was 53.67 (SD = 11.49). A ROC curve analysis was performed to evaluate the sensitivity and specificity of the APACHE II and SAPS 3 tools (Figure 2).

The results showed that both APACHE II (area under the curve [AUC] = 0.854, 95% confidence interval [CI]: 0.795-0.912; sensitivity = 81%; specificity = 75%) and SAPS 3 (AUC = 0.761, IC: 0.686-0.835; sensitivity = 75%; specificity = 64%) were statistically significant in predicting mortality in cardiac surgery, with optimal cutoff points at 14/15 and 46/47, respectively, indicating that patients with higher scores had a greater likelihood of death.

DeLong’s test reveals that the APACHE model (AUC = 0.854) demonstrates excellent discriminative ability to predict the mortality outcome, outperforming the SAPS model (AUC = 0.761), which, although performing well, has inferior performance. The difference between the models is statistically significant (*P* = 0.0058, *P* < 0.05), corroborated by the CI of the difference between the AUCs (0.027 to 0.159), which does not include zero, confirming the superiority of the APACHE model over the SAPS in predicting the outcome.

Therefore, the APACHE II tool proved to be more sensitive and specific as a predictor of mortality in patients who underwent cardiac surgery compared to SAPS 3, although both are good tools for predicting mortality.

A binary logistic regression (using the enter method) was performed to investigate the extent to which mortality could be predicted by the following comorbidities: non-insulin-dependent diabetes, insulin-dependent diabetes, COPD, alcoholism, chronic atrial fibrillation, hypertension, non-dialysis-dependent chronic renal insufficiency, smoking, obesity, chronic coronary insufficiency, and type of surgery.

The model showed sensitivity [χ^^2^^ (12) = 36.910, *P* < 0.001; Nagelkerke R^2^ = 0.155] and was able to adequately predict 88% of the cases (Table 1). Among all predictors, only COPD, non-dialysis-dependent chronic renal insufficiency (non-dialysis chronic kidney disease [ND-CKD]), and the type of surgery had a statistically significant impact, increasing the OR of mortality. Specifically, having COPD increased the OR of mortality by 9.4 times, ND-CKD increased it by 6.4 times, and the type of surgery, in the case of emergency surgery, increased it by four times compared to elective surgery (Table 2).

**Table 1:** Study population data.

Variable	Total (%)	Mortality (%)
	(n = 433)	(Total = 54)
Sex		
Male	263 (59)	33 (61)
Female	170 (39)	21 (39)
Non-insulin-dependent diabetes		
No	368 (85)	48 (13)
Yes	65 (15)	6 (9)
COPD		
No	427 (98)	3 (0,70)
Yes	6 (2)	3 (50)
Alcoholism		
No	398 (92)	47 (12)
Yes	35 (8)	7 (20)
Chronic atrial fibrillation		
No	395 (91)	47 (12)
Yes	38 (9)	7 (18)
Hypertension		
No	212 (49)	22 (10)
Yes	221 (51)	32 (14)
Non-dialysis-dependent chronic renal insufficiency		
No	425 (98)	50 (12)
Yes	8 (2)	4 (50)
Type of surgery		
Elective surgery	375 (87)	37 (10)
Emergency surgery	58 (13)	17 (29)
Smoking		
No	346 (80)	40 (12)
Yes	87 (20)	14 (16)
Obesity		
No	408 (94)	51 (12)
Yes	25 (6)	3 (12)
Chronic coronary insufficiency		
No	342 (79)	47 (14)
Yes	91 (21)	7 (8)
Insulin-dependent diabetes		
No	423 (98)	54 (12)
Yes	10 (2)	0 (0)

COPD=chronic obstructive pulmonary disease

**Table 2:** Predictor variables of mortality.

	Wald	df	Sig.	Exp (B)	95% CI to Exp (B)	
					Lower	Upper
Insulin-dependent diabetes	0	1	0,999	0	0	000.0
Non-insulin-dependent diabetes	2,25	1	0,134	0,454	0,161	1,274
COPD	4,816	1	0,028	9,417	1,271	69,784
Alcoholism	0,27	1	0,603	1,336	0,449	3,978
Chronic atrial fibrillation	0,356	1	0,55	1,354	0,501	3,66
Hypertension	2,817	1	0,093	1,751	0,91	3,368
ND-CKD	4,272	1	0,039	6,48	1,101	38,127
Chronic heart failure	2,677	1	0,102	0,471	0,192	1,16
Obesity	0,139	1	0,71	0,773	0,199	2,998
Smoking	0,142	1	0,706	1,168	0,522	2,613
Type of surgery	14,591	1	0	4,044	1,975	8,284
Sex	0,06	1	0,806	1,083	0,571	2,058
Constant	0	1	0,999			

CI=confidence interval; COPD=chronic obstructive pulmonary disease; df=degrees of freedom; Exp (B)=exponential B; ND-CKD=non-dialysis chronic kidney disease; Sig=Significance

## DISCUSSION

The results demonstrate that the APACHE II tool can be an efficient prognostic index or score with good discriminatory power regarding the prediction of mortality outcomes in non-surviving patients admitted to the ICU and undergoing cardiac surgery, in contrast to survivors. Furthermore, APACHE II more accurately identified patients who would die (85%) compared to the SAPS 3 scoring model (76%). The APACHE II score was incorporated into the assessment of severity and patient status in ICUs by the Brazilian Ministry of Health in 1998^[^8^]^.

In the study by Yalçin et al. (2019)^[^14^]^, with a sample population of 600 ICU patients undergoing cardiac surgery and a mean age of 64.77 years, the prediction score determined by the AUC of APACHE II was 0.743, considering a cutoff value > 9, which enhanced sensitivity (56.25) and specificity (83.58). In the present study, with a total population of 433 ICU patients after cardiac surgery and a mean age of 61 years, the calculated AUC prediction score was 0.853, considering a cutoff value of 14/15, which enhanced sensitivity (81%) and specificity (75%), that is, with an AUC score approximately 14.8% higher. Even considering that the exclusion of patients who died in less than 24 hours can distort the results towards better predictive performance. In this context, the reference for ROC curve prediction scores is standardized, with scores calculated by AUC between 0.7 and 0.8 classified as acceptable, scores between 0.8 and 0.9 categorized as very good, and scores above 0.9 considered excellent.

In the evaluation of the efficacy of SAPS 2 and SAPS 3 scores for patients undergoing cardiac surgery between January 2007 and December 2010, a prospective study conducted with 5,207 patients at a university hospital located in Muenster (Germany) concluded that both models (SAPS 2 and SAPS 3) are not reliable for predicting population mortality after cardiac surgery. Considering the first day of interaction, the AUC results found were 0.804 and 0.757 for SAPS 2 and SAPS 3, respectively^[^11^]^. In the present analysis, the calculated AUC result for SAPS 3 was 0.763, approximately 0.79% higher. Additionally, it is worth mentioning that no data on the SAPS 2 score was collected and processed for correlation.

The variables used as a source of information for calculating the severity index and, consequently, the risk of mortality outcomes in critically ill patients differ between APACHE II and SAPS 3. In the case, APACHE II collects data during the first 24 hours of hospitalization, while SAPS 3 uses data only from the first hour after ICU admission and does not take into account physiological variables or postoperative clinical condition. Additionally, for both APACHE II and SAPS 3, part of this information comes from the patient’s medical history and clinical condition. Furthermore, exclusively for SAPS 3, demographic variables involving patients admitted to the ICU are also considered^[^8^,^14^]^.

Although EuroSCORE II is a specific and easily applicable tool recommended by Brazilian guidelines for preoperative mortality risk assessment in cardiac surgery, particularly for valvular disease, its main limitation compared to APACHE II and SAPS 3 is that it relies solely on preoperative data and does not account for physiological variables or the patient’s postoperative clinical condition. In contrast, APACHE II and SAPS 3, while more complex, provide a more comprehensive evaluation of critically ill patients by incorporating clinical and laboratory parameters collected within the first 24 hours (APACHE II) or the first hour (SAPS 3) of ICU admission. This allows for a more accurate real-time assessment of disease severity and prognosis.

It is crucial to recognize that EuroSCORE II is essential for preoperative planning and decision-making. On the other hand, APACHE II is a fundamental tool for postoperative management in the ICU, quantifying the true impact of surgery on the patient's physiological state and guiding IC. It is worth highlighting that APACHE II can complement specific cardiac models (*e.g.*, EuroSCORE II) for postoperative monitoring and, when used together, provide a comprehensive view of the patient's journey from surgical indication to recovery.

APACHE II also takes into account 12 physiological and laboratory variables directly associated with the risk of mortality, in addition to the patient’s age and chronic health status, requiring an initial diagnosis. On the other hand, SAPS 3 evaluates 20 variables, including physiological, laboratory, demographic, and reasons for ICU admission, without requiring a primary diagnosis. However, while APACHE II emphasizes the assessment of the patient’s chronic health, SAPS 3 focuses on analyzing the patient’s health status prior to hospital admission^[^7^,^8^,^14^,^15^]^.

Evaluating the findings of a single-center, prospective study with a sample of 58 patients with hepatic cirrhosis undergoing cardiac surgery, the scores of different prognostic scoring systems for critically ill patients, including APACHE II and SAPS 3, commonly used in ICUs, were assessed. The SAPS 3 score showed acceptable results for sensitivity (71.4%) and specificity (73.5%), considering a cutoff value of 14.8 ± 8.0. In contrast, the APACHE II score performed worse than the SAPS 3 model and was not considered an efficient tool^[^16^]^.

In a study conducted at a German teaching hospital with a cohort of 1,851 surgical patients admitted to the ICU, with a predominance of male patients (63.4%) compared to female patients (36.6%) and an in-hospital mortality rate of 9%, with a higher proportion of cardiac surgery patients (26.4%) compared to other groups, a comparison of performance was made using the ROC curve for the mortality prediction indices SAPS 2 (0.83), SAPS 3 (0.89), and APACHE II (0.78). The results regarding the discriminatory potential of the scores indicate similar performance in all cases, with a slight emphasis on SAPS 3, as the calculation of the score obtained by patients can be performed within the first hour of ICU admission^[^13^]^. In contrast, the results obtained in this study, with a predominantly male population (59%) and an in-hospital mortality rate of 12.5%, are opposite, with the AUC prediction score of APACHE II (0.853) being higher than that of SAPS 3 (0.763).

However, it is relevant to assess the organic health of post-surgical cardiac patients using ICU scores, disregarding transient adverse conditions, including biochemical and hemodynamic changes in patients, influenced by life-support devices during procedures, such as extracorporeal circulation, intra-aortic balloon pump, and ventricular assist devices (VAD)^[^5^,^12^]^.

In the prospective and observational study by UĞUR et al. (2023)^[^12^]^, which evaluated 204 patients undergoing exclusively CABG, it was concluded that the Cardiac Surgery Score (or CASUS) had superior efficacy in predicting mortality and ICU length of stay compared to APACHE II at seven and 30 days. The sample was predominantly composed of men (n = 166), with a mean age of 60.5 ± 0.7 years, and the overall mortality rate at 30 days was 4.9% (n = 10). Although the present study did not exclusively include CABG patients, they were part of the sample population.

Various etiological factors can influence the development and/or progression of cardiovascular diseases, resulting in complications such as myocardial infarction, cardiac arrhythmias, stroke, or even, in more severe cases, leading to mortality^[^3^]^. In this context, based on the evaluation of comorbidities as a method of predicting mortality in the population of cardiac surgery patients, only two influence the probability of mortality outcomes: COPD and ND-CKD.

Coinciding with this scenario, COPD, responsible for the third leading cause of death (2019), represents a risk factor for the development of concomitant cardiovascular diseases^[^17^-^19^]^. In turn, patients affected by COPD have a two to five times higher risk of developing heart diseases, including: heart failure (HF), ischemic heart disease, arrhythmias, and/or peripheral vascular disease^[^19^]^. A study analyzing 2,500 adult patients with COPD, aged > 40 years, identified an increased probability of developing cardiovascular diseases across all phenotypes, including: HF, coronary heart disease, stroke, and myocardial infarction^[^20^]^.

The findings of Graul et al. (2024)^[^21^]^ and Daniels et al. (2024)^[^22^]^ with samples of over 100,000 COPD patients who experienced ≥ 1 exacerbation (a worsening of disease symptoms), concluded that the risk of mortality and adverse cardiovascular events remained elevated from one to 30 days after the episode, varying in intensity and decreasing over time, yet persisting for up to one year (mortality) and two years (cardiovascular events). Additionally, subsequent exacerbations in patients were associated with gradually higher rates of death and cardiovascular events. The results of this research indicate a 9.4 times higher risk of mortality for patients undergoing cardiac surgery in the evaluated sample.

Based on the results of this study, CKD increased the likelihood of mortality among cardiac surgery patients by 6.4 times. CKD acts as an inducer of cardiotoxicity, physiological dysfunctions, anemia, malnutrition, elevated blood pressure, and HF. Moreover, as renal function declines, the probability of HF occurrence can exceed 65%, reaching 70% in advanced cases^[^23^]^. Iwanaga et al. (2010)^[^24^]^ reported that patients with CKD have a 500 times higher probability of death compared to healthy individuals. Additionally, it is mentioned that a decrease in the glomerular filtration rate of 10 ml/min corresponds to a 7% increase in the frequency of deaths^[^24^]^.

Furthermore, the binary logistic regression highlighted that the predictor of emergency cardiac surgery increased the likelihood of patient mortality by four times compared to elective surgery. Similarly, Gomes et al. (2007)^[^25^]^ argue that the number of deaths among cardiac surgery patients is quadrupled in the case of emergency surgeries in the United Kingdom.

The findings of a retrospective study conducted in Brazil with patients undergoing CABG highlight a higher in-hospital mortality rate in emergency surgery patients (36.4%), followed by urgent surgery patients (16.3%), and finally elective surgery patients (7.0%)^[^26^]^. Thus, although the patient sample in the present study is not exclusively composed of CABG patients, a fraction of them is included in the sample for correlation.

No statistically significant results were found regarding the other predictors (non-insulin-dependent diabetes, insulin-dependent diabetes, alcoholism, chronic atrial fibrillation, hypertension, smoking, obesity, chronic coronary insufficiency, and type of surgery) in predicting post-surgical cardiac mortality in the described sample population. The literature evaluating the efficacy of the APACHE II and SAPS 3 mortality prediction indices, either jointly in the same study or separately in different studies, specifically for populations of patients after cardiac surgery, is scarce. Therefore, we emphasize the relevance of the present study.

### Limitations

Among the limitations of this study, we highlight its retrospective observational design, the relatively small cohort of 433 patients, and the fact that data were collected in a single institution. Although the discrimination analysis (AUC) demonstrated robust performance, the calibration of the model (Hosmer-Lemeshow test) was not assessed. Therefore, further prospective studies with larger samples are needed to confirm the ability of APACHE II and SAPS 3 to predict mortality in critically ill patients admitted to the ICU. In this regard, multicenter investigations involving different hospitals may yield more reliable and generalizable results, as they incorporate diverse patient populations and ICU settings, thereby reducing the potential influence of local healthcare practices.

## CONCLUSION

The predictive indices APACHE II and SAPS 3 scores are competent tools for predicting the risk of mortality outcomes in cardiac surgery patients admitted to the evaluated ICUs. However, the APACHE II prognostic score demonstrated superior performance in predicting mortality outcomes in cardiac surgery patients admitted to the ICU, showing higher sensitivity and specificity compared to SAPS 3, and consequently, greater discriminatory power. The use of prognostic indices such as APACHE II ensures that physicians, patients, and families make informed decisions regarding treatment, considering the hospitalized patient’s health condition, resulting in the provision of excellent services by hospital institutions.

## Data Availability

The authors declare that they cannot share data on hospitalized patients due to the need to maintain confidentiality and privacy.

## Artificial Intelligence Usage

The authors declare that no artificial intelligence tool was used in the preparation of this article.
